# The Preparation of {001}TiO_2_/TiOF_2_ via a One-Step Hydrothermal Method and Its Degradation Mechanism of Ammonia Nitrogen

**DOI:** 10.3390/ma15186465

**Published:** 2022-09-17

**Authors:** Jiaming Zhu, Zuohua Liu, Feiyun Yang, Dingbiao Long, Yue Jian, Shihua Pu

**Affiliations:** 1 Chongqing Academy of Animal Sciences, Chongqing 402460, China; 2 National Center of Technology Innovation for Pigs, Chongqing 402460, China; 3 Scientific Observation and Experiment Station of Livestock Equipment Engineering in Southwest, Ministry of Agriculture, Chongqing 402460, China

**Keywords:** {001}TiO_2_/TiOF_2_, ammonia nitrogen, photocatalysis, mechanism

## Abstract

{001}TiO_2_/TiOF_2_ photocatalytic composites with a high activity {001} crystal plane were prepared by one-step hydrothermal methods using butyl titanate as a titanium source and hydrofluoric acid as a fluorine source. X-ray diffraction (XRD), scanning electron microscopy (SEM), high-resolution transmission electron microscopy (HRTEM), raman spectroscopy, N_2_ adsorption-desorption curve (BET), UV-Vis diffuse absorption spectroscopy (UV-Vis DRS), X-ray photoelectron spectroscopy (XPS), and fluorescence spectroscopy (PL) were used to evaluate the structure, morphology, specific surface area, optical properties, and photocarrier separation ability of {001}TiO_2_/TiOF_2_. Ammonia nitrogen was taken as the target pollutant, and the degradation performance of the catalyst was investigated. The results show that hydrofluoric acid improves the content of {001} crystal plane of TiO_2_ with high activity; it also improves the specific surface area and dispersion of the composite material and adjusts the ratio of {001}TiO_2_ to TiOF_2_ in the composite material to enhance the absorption capacity of the composite material and reduce the band gap width of the composite material. The degradation rate of ammonia nitrogen by 100 mg F15 is 93.19% when the initial concentration of ammonia nitrogen is 100 mg/L and pH is 10. Throughout the reaction process, the {001}TiO_2_/TiOF_2_ composite produces superoxide anion radical (·O_2_^−^) and hydroxyl radical (·OH) to oxidize NH_3_·H_2_O and generate N_2_ accompanied by a small amount of NO_3_^−^ and NO_2_^−^.

## 1. Introduction

Ammonia nitrogen is one of the main forms of inorganic nitrogen in water; it is toxic to aquatic animals and plants and consumes dissolved oxygen. It is also a nutrient leading to the eutrophication of water bodies [1,2,3]. If the nitrate nitrogen converted from ammonia nitrogen is absorbed by infants through drinking water, then it can cause methemoglobin disease. At the same time, nitrosamines with “triple action” are generated when ammonia nitrogen is oxidized to nitrite nitrogen; these can threaten human health [4]. Nitrification, denitrification, and anammox are usually used to remove ammonia nitrogen and total nitrogen [5,6]. They are easily affected by environmental temperature and pollutant components, and denitrification efficiency is unstable. The treatment process takes a long time. As a result, it is urgent to develop new efficient and clean technologies.

Alshorif et al. reported quantum dots supported by cesium selenide nanocomposites to effectively degrade rhodamine B [7]. They further used CS-Au /MgFe_2_O_4_ to catalyze the degradation of 3 and 4 dihydropyrimine 2(1H) and RhB dyes with degradation rates of 99.0 and 96.68%, respectively [8]. In the direction of energy synthesis, a simple synthesis of phosphotungstate acid (PTA) with different weight contents (10, 20, 25, 50, and 75%) on UIO-66 (Zr) was also reported as a highly efficient and reusable catalyst for the preparation of coumarin and dihydropyrimidine derivatives [9]. Photocatalysisoffers high efficiency, energy savings, and environmental friendliness. It can overcome problems based on biological methods and is one of the most promising pollutant treatment technologies [10,11]. John et al. [12] reported the degradation of polychlorophenol by TiO_2_, which initiated research into semiconductor photocatalytic materials in water pollution treatment. However, its application is limited due to the low utilization rate of TiO_2_ for light and the easy recombination of photogenerated carriers. Studies showed that TiO_2_ could be recombined with other semiconductor materials to form heterogeneous structures to reduce the band gap width of TiO_2_, improve its photogenerated carrier separation ability, and improve its degradation performance [13]. For example, Zhou et al. [14] used B-SiO_2_/TiO_2_ composite material to degrade ammonia nitrogen by 65% (43 mg/L, pH = 8) at 660 min. Yao et al. [15] used Cu-H_3_PW_12_O_40_ /TiO_2_ composite material to degrade ammonia nitrogen by 80% (300 mg/L, pH = 11) at 420 min. Li et al. [16] used g-C_3_N_4_ /rGO/TiO_2_ composite material to degrade ammonia nitrogen to 95% (50 mg/L, pH = 9.5) at 240 min. These studies show that it is feasible to combine other photocatalytic materials with TiO_2_ to form heterogeneous structures for ammonia nitrogen degradation. Nevertheless, the complex preparation process increases the difficulty and cost of photocatalytic materials synthesis, and materials with a simple preparation method and good catalytic activity under sunlight are of urgent interest.

Li et al. [17] showed that fluoride ions could replace oxygen atoms in TiO_2_ lattice and directly connect with titanium atoms, thus generating new TiOF_2_. Zhang et al. [18] found that TiO_2_/TiOF_2_ composite photocatalytic material had better activity than single TiO_2_ and TiOF_2_ because the band gap width of TiOF_2_ was about 2.6–2.8 eV (much smaller than 3.12 eV of TiO_2_). The photocatalytic activity and photoquantum efficiency of TiO_2_ can be greatly improved, and the defects of TiO_2_ can be made up by effectively utilizing the visible light part with more content in the sunlight and the advantages of a short photogenerated carrier migration path.

There are currently only a few reports showing that TiO_2_/TiOF_2_ can be directly prepared via a one-step hydrothermal method, and most studies prepare TiO_2_/TiOF_2_ composite photocatalytic materials by high-temperature treatment of TiOF_2_ precursor. For example, such as Wang et al. [19] obtained TiO_2_/TiOF_2_ by calcination of cubic TiOF_2_ precursor at 500−600 °C for 2 h. Huang et al. [20] believed that 500−600 °C was highly wasteful of energy, and TiOF_2_ was transformed in situ at 180 °C with alcohol as a solvent, thus forming TiO_2_/TiOF_2_. In this paper, TiO_2_/TiOF_2_ composite catalyst with high activity {001} crystal surface was prepared by a one-step hydrothermal method. By using ammonia nitrogen as the target pollutant, the degradation of ammonia nitrogen by {001}TiO_2_/TiOF_2_ under sunlight was achieved for the first time. This process provided the corresponding theoretical basis for ammonia nitrogen treatment.

## 2. Methods

### 2.1. Preparation of Catalysts

Hydrofluoric acid (0, 3, 6, 9, 12, 15, and 20 mL) was slowly dropped into 51 mL butyl titanate at a rate of 2–3 drops per second, and then 90 mL CH_3_COOH was slowly added at the same rate, the precursors were then separated and dispersed using a cell fragmentation apparatus (Ningbo, Zhejiang Province, China. Xinzhi Biotechnology Co., Ltd., SCIENTZ-IID, 65 Hibiscus Road, Ningbo National High-tech Zone.). The mixed solution was stirred at a constant speed for 30 min at room temperature and then transferred to a stainless-steel reactor containing a Teflon liner and reacted at 180 °C for 15 h under high pressure. After cooling to room temperature, the product was cross-washed with ultrapure water and absolute ethanol three times, dried in a 60 °C air drying oven, and then ground. The product was abbreviated as FX, namely, F0, F3, F6, F9, F12, F15, and F20.

### 2.2. Catalyst Characterization

X-ray diffractometer (XRD, Bruker, Mannheim, Germany) model D8 Advance was used to test the crystal characteristics of the samples. The operating parameters were X-ray tube target = Cu. Jsm-6700 scanning electron microscope (SEM, Tokyo, Japan, JEOL) was used to observe the morphology and dispersion of the samples. Tecnai G2 20 transmission electron microscope (TEM, FEI, Hillsboro, OR, USA) was used to observe the lattice spacing of the samples. HR800 laser confocal Raman spectrometer (Raman, Horiba Jobin Yvon, Palaiseau, France) was used to test the crystal surface content of the samples, and the excitation wavelength was 633 nm. A series of automatic surface analyzer ASAP 2460 (BET, Norcross, GA, USA) was used to determine the specific surface area, pore volume, and pore diameter of the catalyst. The samples were pretreated with vacuum degassing at 200 °C for 5 h, and high purity nitrogen was used as adsorbent at 77K. A Tensor 27 Fourier transform infrared spectrometer (FT-IR, Bruker, Mannheim, Germany) was used to test the surface functional groups of the catalyst at a resolution of 2 cm^−1^. X-ray photoelectron spectroscopy (XPS, Thermo, Waltham, MA, USA) EscaLab 250Xi was used for elemental and valence analysis, and binding energy C1s (284.6 eV) of carbon adsorbed on the surface were used as an internal standard to calibrate the binding energy. Hitachi F-7100 fluorescence spectrophotometer was used to test the photogenerated carrier separation ability of the samples, and a hydrogen light source with a pulse width of 1.0–1.6 nanoseconds was used to test the fluorescence lifetime of the samples. Dynamic light scattering (DLS, Mastersizer2000, Malvern Panalytical, Malvern, UK) was used to test the particle size distribution of materials, ranging from 0.02 nm to 2000 µm. The 5,5-dimethyl-1-pyrrolidine N-oxide (DMPO) was used as the spin trapping agent for ESR analysis.

### 2.3. Photocatalytic Activity Tests

Ammonium chloride was used to prepare 100 mL ammonia nitrogen solution with different concentrations, and the pH was adjusted by hydrochloric acid and sodium hydroxide—these were added to the reactor with different amounts of FX. After turning on (or off) the xenon lamp, 2 mL reaction liquid sample was extracted every 1 h and centrifuged for 10 min in a 10,000 r/min high-speed centrifuge. The supernatant was used to determine the concentration of ammonia nitrogen and calculate its degradation rate *η* (Equation (1)). The concentrations of NO_3_^−^ and NO_2_^−^ after the reaction were then determined.

*η* = (*C_0_ − C*_1_) × 100%/*C*_1_ (1)

where *C*_0_ is the initial concentration of ammonia nitrogen, *C*_1_ is the concentration of ammonia nitrogen in the reaction process, mg/L.

## 3. Results and Discussion

### 3.1. Analysis of Characterization Results

#### 3.1.1. XRD Analysis

**Figure 1:**
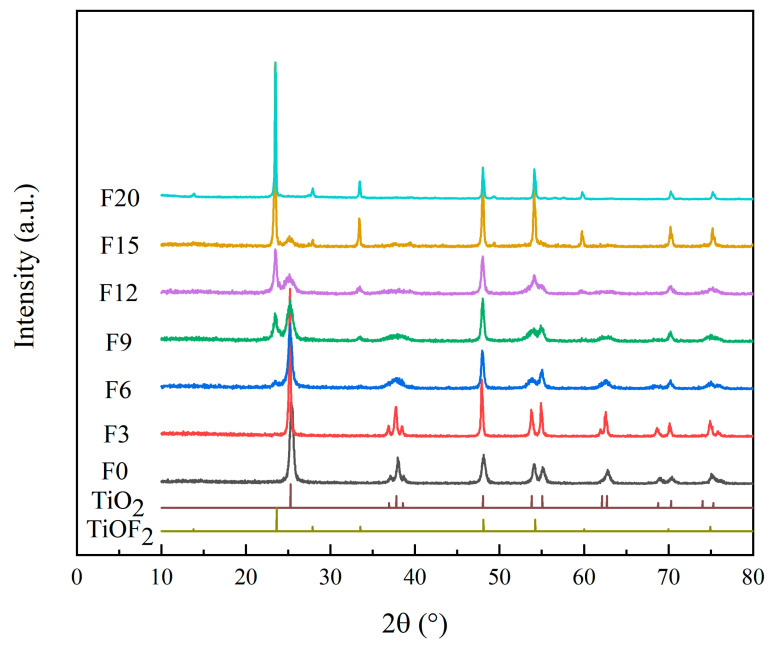
XRD patterns of samples.

We found 2θ = 25.28, 36.94, 37.80, 38.57, 48.04, 53.89, 55.06, 62.68, 70.31, and 75.03°, which correspond to anatase TiO_2_ (JCPDS No. 21-1272) [21], and 2θ = 13.61, 23.39, 27.68, 33.41, 47.83, 53.89, 59.59, 69.58, and 74.68°, are largely in line with TiOF_2_ (JCPDS No. 01-0490) [22]. Figure 1 shows the XRD pattern of the prepared samples. Only TiO_2_ characteristic peaks appear in F0 and F3, thus indicating that they are pure TiO_2_. Only the TiOF_2_ characteristic peaks appear in F20, thus indicating that they are pure TiOF_2_. The F6, F9, F12, and F15 show not only TiO_2_ characteristic peaks but also show TiOF_2_ characteristic peaks, indicating that they are TiO_2_/TiOF_2_ composite photocatalytic samples. Hydrolysis and polymerization occur in the process of material preparation [23] (Equations (2)–(4)). In the polymerization stage, when HF is absent in the solution system or its concentration is too low, fluorine atoms cannot enter the TiO_2_ lattice to replace O atoms, and the final products of F0 and F3 can only be pure TiO_2_. Fluorine atoms gradually enter the TiO_2_ lattice to replace O atoms when the amount of HF gradually increases. This explains why F6 is dominated by TiO_2_ and contains trace TiOF_2_ while F15 is dominated by TiOF_2_ and contains trace TiO_2_. The HF content plays a decisive role in the final product. Here, only TiO_2_/TiOF_2_ composite photocatalytic materials were studied; F0, F3, and F20 were not studied.

Ti(OC_4_H_9_)_4_ + H_2_O → Ti(OH)_4_ + 4(C_4_H_9_OH) (hydrolysis) (2)

Ti(OH)_4_ + 2HF → TiOF_2_ + 3H_2_O (Polymerization) (3)

Ti(OH)_4_ + Ti(OH)_4_ → 2TiO_2_ + 4H_2_O (Polymerization) (4)

#### 3.1.2. Morphology and Lattice Spacing Analysis

**Figure 2:**
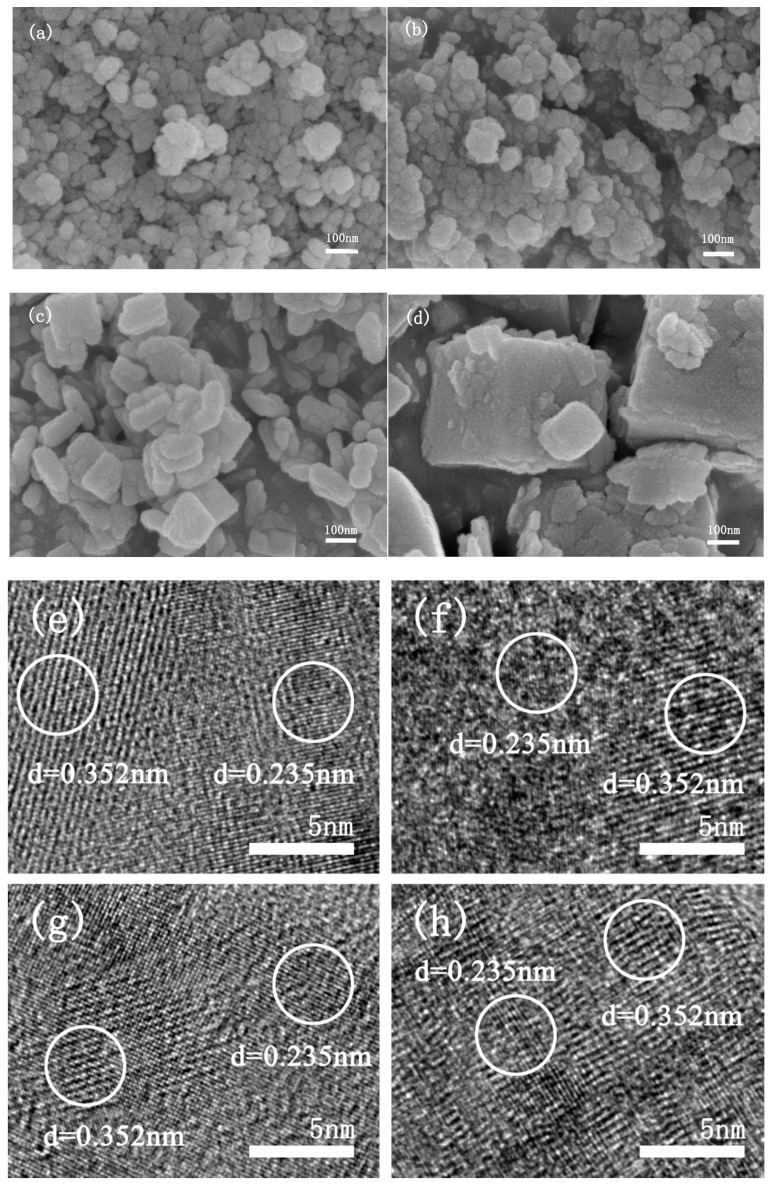
SEM of F6 (**a**), F9 (**b**), F12 (**c**), F15 (**d**), HRTEM of F6 (**e**), F9 (**f**), F12 (**g**), F15 (**h**).

Figure 2 shows that F6 and F9 have very similar morphologies—both are accumulated by small irregular pieces with poor dispersion. F12 has a thin section with good dispersion, and F15 is a mixture of regular large pieces and small irregular pieces with good dispersion. In HRTEM (Figure 2e–h), the spacing of the lattice fringe was 0.352 nm and 0.235 nm, respectively, corresponding to the lattice distance of the {101} and {001} plane of anatase TiO_2_. The decreasing order of the surface energies of anatase TiO_2_ is 0.90 J/m^2^ for {001}, 0.53 J/m^2^ for {100}, and 0.44 J/m^2^ for {101} [24]; most anatase TiO_2_ has a low-energy {101} surface rather than high-energy {001} facets [25]. However, due to the presence of F ion in the preparation process, the energy of {001} crystal plane can be reduced. Therefore, we found that the prepared FX contains {001} crystal plane, namely {001}TiO_2_/TiOF_2_.

#### 3.1.3. Raman Analysis

**Figure 3:**
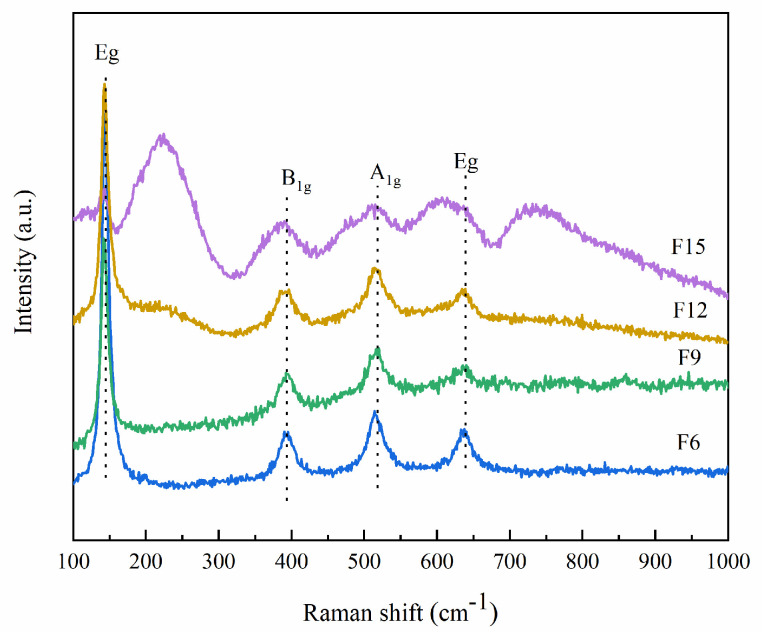
Raman patterns of samples.

Anatase TiO_2_ Raman spectra have six bands: their frequencies are A1g (*ν* = 514 cm^−1^), B1g (*ν* = 402 cm^−1^ and 522 cm^−1^) and Eg (*ν* = 144 cm^−1^, 197 cm^−1^ and 635 cm^−1^). Anatase is teteal and the space group is I41/AMD (D19/4H); thus, the bands *ν* = 197 cm^−1^ and 522 cm^−1^ are the weakest [26]. Figure 3 shows the Raman diagram of the prepared materials. F6, F9, F12, and F15 all contain TiO_2_ bands. By comparison, Hou et al. [27] found that the Raman spectra of TiOF_2_ are at *ν* = 230 cm^−1^ and 750 cm^−1^. Figure 1 shows that the increase in HF content causes the Raman spectrum of TiOF_2_ to become gradually obvious. In particular, F12 and F15 have a better Raman spectrum of TiOF_2_. This is because the Raman spectrum of TiOF_2_ is weaker than TiO_2_, and its characteristic spectrum cannot be displayed when the content is not high enough.

In addition, Tian et al. [28] calculated the content of the prepared catalyst {001} surface by Raman; this is the ratio of the band intensity at A1g (= 514 cm^−1^) to the band intensity at Eg (= 144 cm^−1^). As shown in Table 1, the {001} surface contents of F6, F9, F12, and F15 are 30.09, 52.09, 55.06, and 92.25%, respectively. Such highly active {001} surfaces are beneficial to the photocatalytic reaction.

#### 3.1.4. BET Analysis

**Figure 4:**
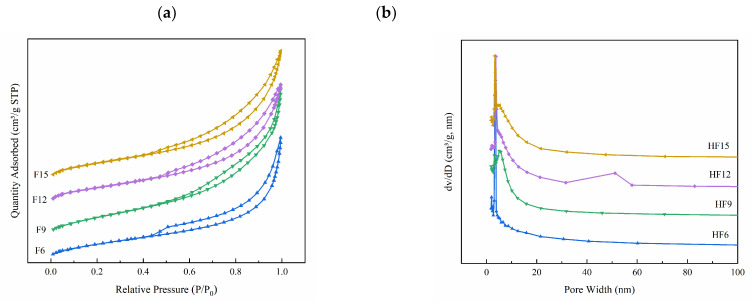
Nitrogen adsorption–desorption curve (**a**) particle size distribution (**b**) of FX.

Figure 4 shows the N_2_ adsorption–desorption curve of the prepared photocatalytic materials and the corresponding pore size distribution of BJH. Figure 4a shows that FX has a typical type IV N_2_ adsorption curve and H_3_ hysteresis regression curve, thus indicating that the catalyst has a mesoporous structure [29]. The pore size distribution in Figure 4b shows that the main pore size distribution of the composite catalyst is within 10 nm, thus indicating that the particle size distribution of the catalyst is narrow. The results of specific surface area and pores of the catalyst calculated by the BJH method are shown in Table 2, respectively. F15 has the largest specific surface area of 60.89 m^2^/g, and there is little difference between pore volume and diameter.

#### 3.1.5. FT-IR Analysis

**Figure 5:**
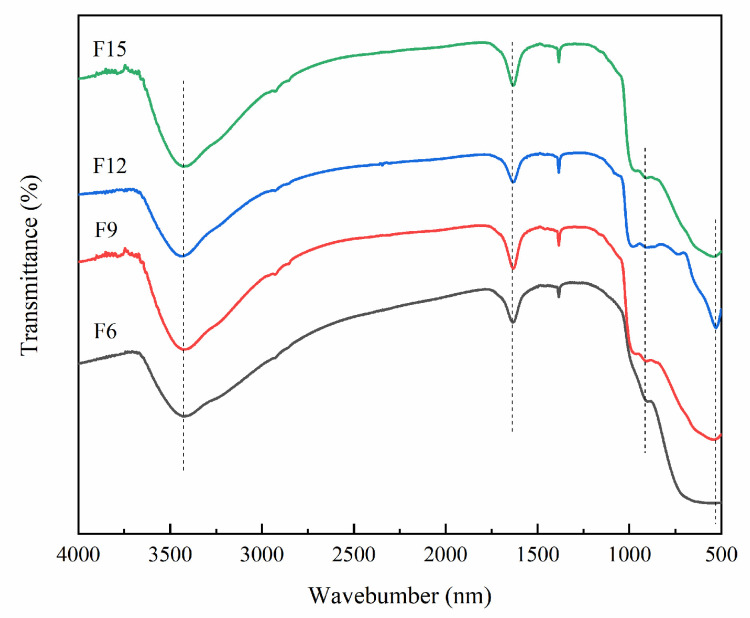
FT-IR diagram of FX.

The surface groups of FX were further confirmed by infrared spectroscopy, and the results are shown in Figure 5. The absorption bands centered at 3429 and 1633 cm^−1^ are caused by hydroxyl radicals and related hydrogen bonds and absorption of water [30]. The absorption band at 922 cm^−1^ was caused by the vibration of Ti–F in TiOF_2_, thus indicating that the F atom replaced part of the oxygen atom in TiO_2_ [31]. The absorption peaks around 500 cm^−1^ are due to the stretching vibration of the Ti–O group [32].

#### 3.1.6. Optical Performance Analysis

**Figure 6:**
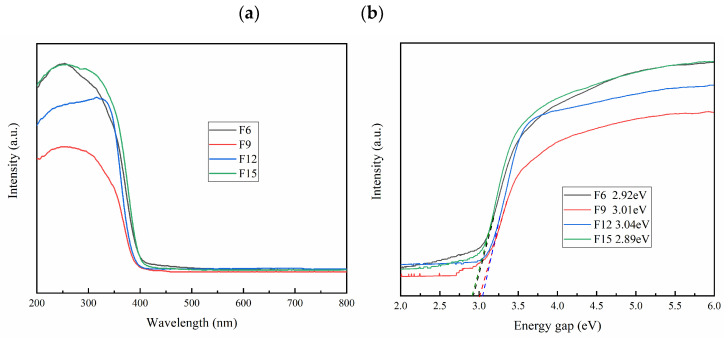
Uv-vis absorption diagram (**a**), band gap (**b**) of the samples.

The UV-vis absorption diagram of FX is shown in Figure 6a. F15 and F6 have the strongest peak in the range of 200–400 nm, thus indicating that it has good absorption of UV. The redshift ability of F15 and F6 is stronger than that of F12 and F15 in the visible region, thus indicating that the utilization rate of light of F15 and F6 is improved, and the ratio between {001} TiO_2_ and TiOF_2_ affects the light absorption performance of the material. The band gap energy (Eg) of the semiconductor is calculated by the Kubelka–Munk function [33]. Combined with Figure 6b, we conclude that F15 has the smallest band gap of 2.89 eV, which indicates that F15 has a narrower band gap and stronger light absorption capacity.

#### 3.1.7. XPS Analysis

**Figure 7:**
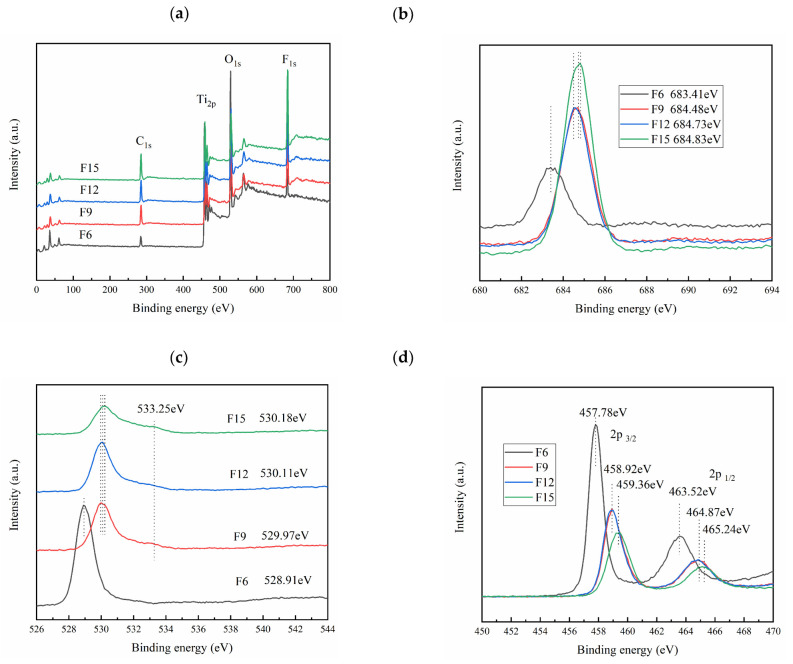
XPS survey spectra for (**a**) all samples, (**b**) F 1s, (**c**) O 1s, and (**d**) Ti 2p of the samples.

XPS is used further to explore the element composition and binding energy of FX. Figure 7a shows that the samples all contain four elements: C, Ti, O, and F. The C comes from the XPS instrument itself, and Ti, O, and F come from TiO_2_/TiOF_2_ samples [34]. Figure 7b shows that the F1s binding energies of F6, F9, F12, and F15 materials are 683.41, 684.48, 684.73, and 684.83 eV, respectively, and their binding energies gradually increase, thus indicating that Ti-F bond on TiO_2_ surface moves to Ti–F in TiOF_2_ lattice [35]. Figure 7c showed that the O1s binding energies of F6, F9, F12, and F15 materials are 528.91, 529.97, 530.11, and 530.18 eV, respectively, thus indicating that the binding energies of O1s and titanium dioxide lattice oxygen (Ti−O−Ti) increase, which may be caused by the replacement of O atoms by more F atoms [36]. In addition, the binding energy of O atoms appears at 533.25 eV for F9, F12, and F15, which corresponds to oxygen vacancy (Ov) and hydroxyl oxygen in adsorbed water [37]. Figure 7d shows that F6 and F9 (material F12) as well as F15 material Ti2p3/2 binding energies are 457.78, 458.92, and 459.36 eV, respectively; F6, F9 (≈F12), and F15 Ti2p1/2 binding energies are, respectively, 463.52, 464.87, and 465.24 eV, This indicates that the binding energy of Ti-F bond is stronger than that of Ti-O bond, indicating that TiOF_2_ is gradually increased in the composite [38]. In addition, Figure 7b–d show that the peaks of F1S of F6, F9, F12, and F15 are gradually enhanced, while the peaks of O1s and Ti2p are gradually weakened, thus further indicating that the proportion of TiOF_2_ in the composite materials is gradually increasing.

#### 3.1.8. PL Analysis

**Figure 8:**
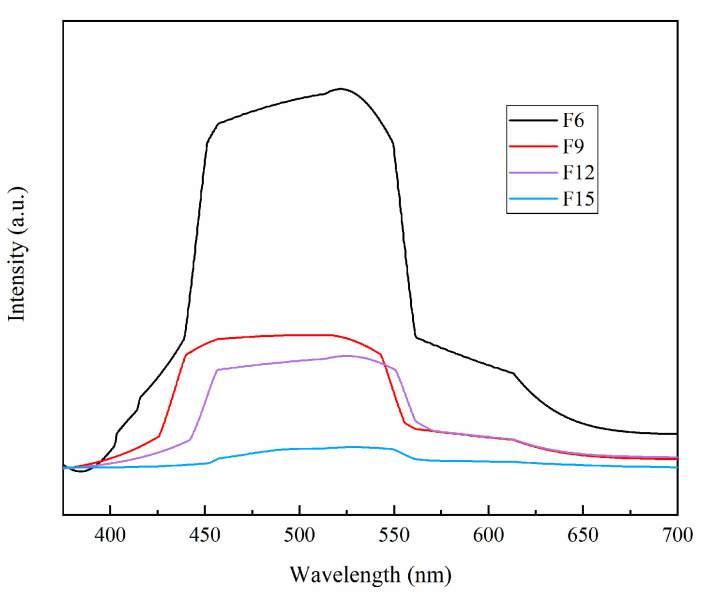
PL diagram of the samples.

The PL spectrum can be used to study the photogenerated carriers’ recombination in semiconductors [39]. A higher peak intensity leads to a higher photogenerated electron–hole recombination rate and a lower photocatalytic activity. The PL spectrum of FX is shown in Figure 8, and F15 has the lowest fluorescence intensity, which indicates that the P-N heterojunction formed by n-type TiO_2_ and p-type TiOF_2_ effectively inhibits the recombination rate of electron–hole pairs and also indicates that the ratio of TiO_2_ and TiOF_2_ in {001}TiO_2_/TiOF_2_ composites is very important.

#### 3.1.9. Particle Size Distribution

**Figure 9:**
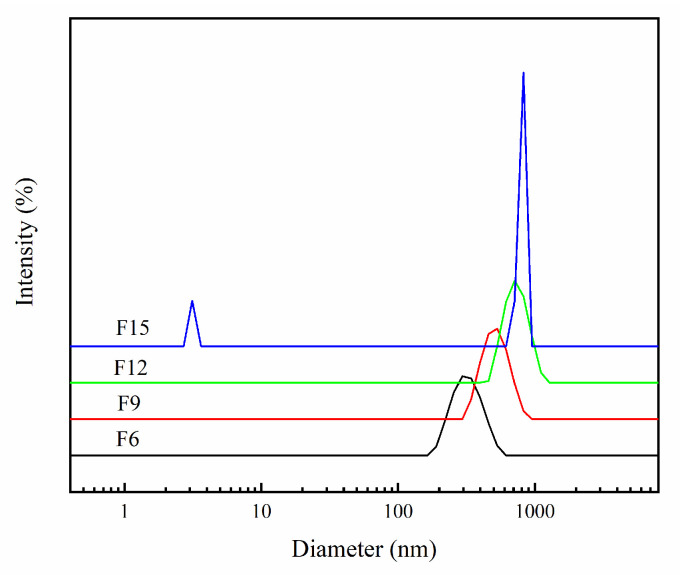
Particle size distribution map of FX.

The particle size distribution of the prepared FX material is shown in Figure 9. The average particle sizes of F6, F9, F12, and F15 are 532.8, 560.8, 837.8, and 1389.0 nm, respectively. When combined with SEM images, we conclude that the average particle size of materials increased gradually as the hydrofluoric acid amount gradually increased. F6 and F9 appear similar—the average particle size is close to F12; F15 significantly increased. F15 has a very weak small peak, and it may be because it dissolves in a water sample; thus, the ultrasonic dispersal time is too long. A few were stripped off and caused a small weak peak in the figure.

### 3.2. Analysis of Photocatalytic Degradation of Ammonia Nitrogen

#### Volatilization of Ammonia Nitrogen

**Figure 10:**
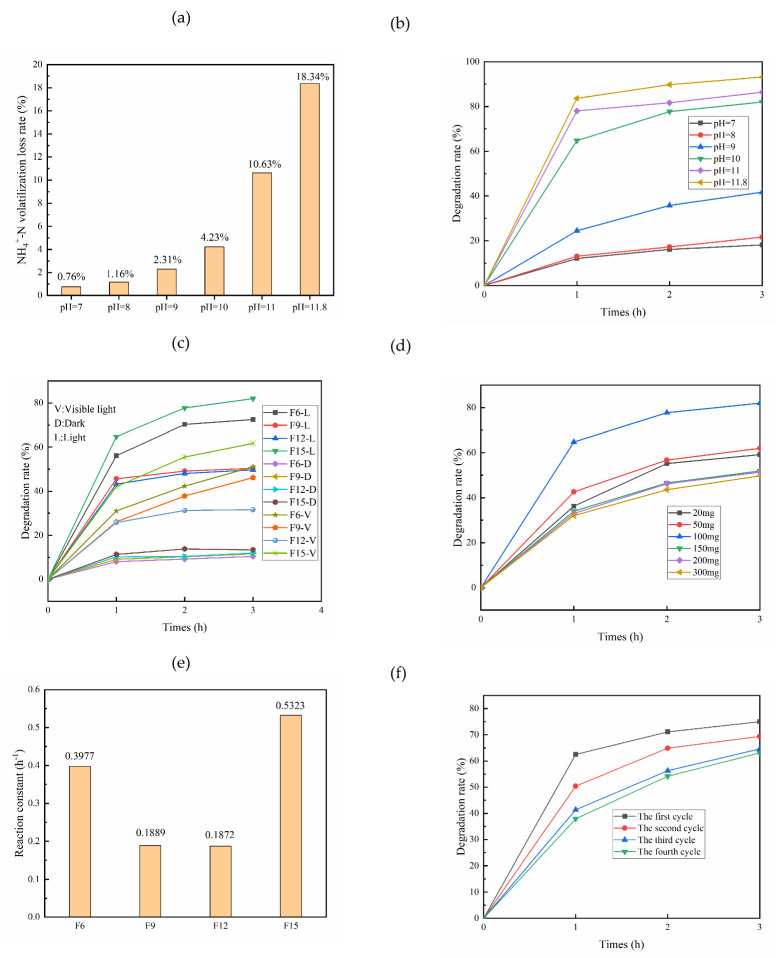
Volatilization loss of ammonia nitrogen at different pH (**a**), effect of pH on ammonia degradation (**b**), effects of different catalysts on ammonia degradation (**c**), effect of catalyst dosage on ammonia degradation (**d**), reaction constant of FX (**e**), repeated degradation property of F15 (**f**).

The volatilization loss of ammonia nitrogen is shown in Figure 10a. The volatilization loss rate of ammonia nitrogen is low, pH < 10, but the loss rate of ammonia nitrogen is 10.63 and 18.34%, respectively, when pH is 11 and 11.8. This is because when pH is > 10.1, ammonia nitrogen in solution mainly exists in the form of NH_3_·H_2_O. When it comes into contact with air, it is easy to transfer from liquid phase to gas phase and can lead to a pungent taste [14].

The initial concentration of ammonia nitrogen was 100 mg/L, and 100 mg of F15 catalyst was added to study its influence on ammonia nitrogen degradation through different pH values. The results are shown in Figure 10b. The ammonia degradation rate increases with increasing pH. When the pH values are 7, 8, 9, 10, 11, and 11.8, the ammonia degradation rates are 18.11, 21.62, 41.71, 81.99, 86.39, and 93.19%, respectively. The evaporation rate is higher when the pH values are 11 and 12. The degradation rate of ammonia nitrogen is not ideal when pH is 7, 8, and 9. The effect on ammonia nitrogen is small, and the degradation rate is high at pH 10. With longer reaction times, the degradation rate of ammonia nitrogen gradually decreases. This is because the concentration of ammonia nitrogen decreases with the progress of the reaction, thus leading to a decrease in the reaction rate.

When the catalyst dosage is 100 mg, the initial concentration of ammonia nitrogen is 100 mg/L, and the pH is 10. With a 300 W xenon lamp (light), ultraviolet light (visible light) was filtered out by JB420 glass, and no light (dark). The corresponding ammonia nitrogen degradation figure is shown in Figure 10c. The ammonia nitrogen removal rate of F6, F9, F12, and F15 is very low when there is no light. These values were 10.43, 11.61, 11.92, and 13.45%, respectively. When UV light was filtered out, the removal rates of ammonia nitrogen by F6, F9, F12, and F15 were 51.12, 46.12, 31.58, and 61.72%, respectively. The degradation rates of ammonia nitrogen by F6 and F15 under visible light were significantly better than those by F9 and F12. This is because the proportion of {001}TiO_2_ and TiOF_2_ in the composite material of F6 and F15 is moderate, and thus their utilization of visible light is high. These data are consistent with optical property characterization. The degradation rate of ammonia nitrogen decreases because the catalyst adsorbs on its own specific surface area. Under xenon lamp irradiation, the degradation rates of ammonia nitrogen are 72.45, 50.38, 49.735, and 81.99%. F6, F9, F12, and F15 are all {001}TiO_2_/TiOF_2_ composite samples, but F15 has the largest specific surface area (60.89 m^2^/g), the highest content of highly active {001} crystal plane (92.25%), the strongest light absorption ability, the strongest photogenerated carrier separation ability, and the smallest bandgap width (2.89 eV). Therefore, F15 not only has the strongest adsorption ability for ammonia nitrogen solution but also has the strongest degradation ability. Wang et al. [22] showed that TiOF_2_-Ref degraded RhB under a 300 W xenon lamp, and the degradation rate was close to 50% when the reaction time was 5 h, and the degradation rate of crushed-TiOF_2_ was close to 80% when the reaction time was 3 h. Huang et al. [20] synthesized TiOF_2_ by microwave-assisted synthesis and found that its activity was very low. The {001}TiO_2_ was obtained by mixing TiOF_2_ and tert-butanol at 180 °C, and its reaction constant was 20-fold that of TiOF_2_. Lv et al. [40] showed that TiO_2_/TiOF_2_ composite catalyst could be generated when TiOF_2_ was calcined at 300 °C. The 375-W high-pressure mercury lamp was used to degrade dye X3B, and its degradation rate was less than 30% at 2 h. This indicates that the strategy of further preparation of TiO_2_/TiOF_2_ by TiOF_2_ is not feasible. The one-step hydrothermal preparation of TiO_2_/TiOF_2_ composite catalysts by controlling the reaction conditions is a potential method.

When the initial concentration of ammonia nitrogen is 100 mg/L and pH is 10, 20, 50, 100, 150, 200, and 300 mg of F15 were added to explore the influence of different dosages on ammonia nitrogen degradation (Figure 10d). The degradation rate of ammonia nitrogen with the catalyst dosing quantity decreases after first rising when the degradation of ammonia nitrogen rate is less than 100 mg. These values are lower when catalyst and ammonia nitrogen cannot fully contact. Very high catalyst dosage affects light utilization and leads to a decrease in the degradation rate.

The reaction constant is an important parameter to measure the degradation performance of photocatalytic materials, which can be calculated by − In (*C*/*C*_0_) = kt, where *C* is the post-reaction concentration, *C*_0_ is the initial concentration, and K is the reaction constant. The larger the value, the stronger the degradation ability. The reaction constant of FX is shown in Figure 10e. F6, F9, F12, and F15 are, respectively, 0.3997, 0.1889, 0.1872, and 0.5323 h^−1^, indicating that F15 has the strongest ability to degrade ammonia nitrogen because the {001} crystal surface of TiO_2_ in F15 has the highest activity content, the largest specific surface area, and the strongest light response in the visible light range. The band gap width is the lowest, and the photogenerated carrier separation ability is the strongest, indicating that F15 has the highest utilization rate of light, so it has the optimal degradation performance.

The repetition performance of F15 is shown in Figure 10f. It can be seen that with the increase in repetition times, its degradation rate decreases. This is because the catalyst needs to be recycled during repeatability testing, and there is a loss with each recovery. We recorded 86, 71, and 53 mg of the catalyst after the first, second, and third recovery, respectively. This means that nearly half of the catalyst is lost after four times being reused. When combined with Figure 10b, It can be seen when the dosage of catalyst is 50 mg, the degradation rate is 62.02%, and the degradation rate is 63.10% after repeated use five times. This indicates that the catalyst is lost in the process of reuse, but its activity remains unchanged.

### 3.3. Mechanism Analysis of Photocatalytic Degradation of Ammonia Nitrogen

**Figure 11:**
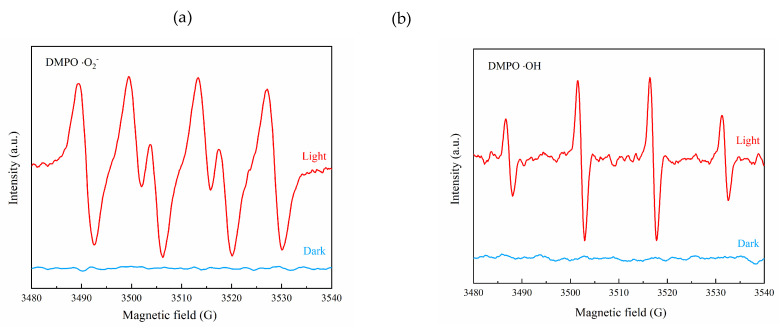
ESR profiles of DMPO-·O_2_^−^ (**a**) and DMPO-·OH (**b**) of F15.

ESR was used to detect the types of free radicals under F15 light and to explore the mechanism of its degradation of ammonia nitrogen. Figure 11a,b shows that F15 did not produce free radicals in the absence of light, and hydroxyl radical (·OH) and superoxide radical (·O_2_^−^) were detected after light. Here, ·OH and ·O_2_^−^ played a decisive role in the entire reaction [31]. The degradation mechanism is given as follows: NH_4_^+^ exists as NH_3_·H_2_O under alkaline conditions. The NH_3_·H_2_O molecules are first adsorbed on the surface of the catalyst. Electrons and holes are generated when the catalyst is exposed to light. They then oxidize water molecules on the surface of the catalyst to form ·OH, while electrons and dissolved oxygen on the surface of the catalyst are formed through a series of reactions to form ·O_2_^−^. The ·OH and ·O_2_^−^ are the main active components in the photocatalytic reaction process via the following process [41]:

{001}TiO_2_/TiOF_2_+hv → {001}TiO_2_/TiOF_2_ (h^+^ + e^−^) (5)

h^+^+ H_2_O → H^+^ + ·OH (6)

e^−^ + O_2_ → ·O_2_^−^ (7)

**Figure 12:**
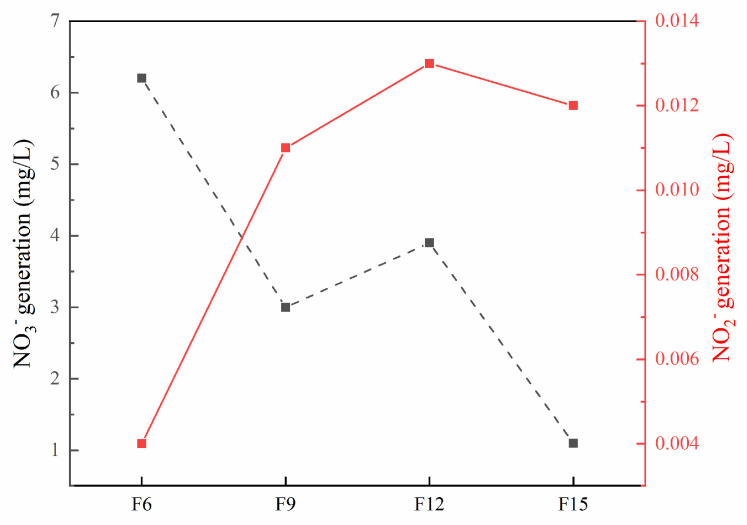
NO_3_^−^and NO_2_^−^ production after the reaction.

In order to further confirm the intermediate products of ammonia nitrogen degradation, the initial concentration of ammonia nitrogen was held at 100 mg/L. Next, 100 mg of F15 is added with pH 10 and a reaction time of 180 min. The results are shown in Figure 12. Here, NO_3_^−^ and NO_2_^−^ concentrations are no more than 6.21 and 0.013 mg/L, respectively. There are three main reaction products of ammonia nitrogen, NO_3_^−^, NO_2_^−^, and N_2_, the low content of NO_3_^−^ and NO_2_^−^ indicates that ammonia nitrogen is mainly oxidized to N_2_. We speculated the following on the process of photocatalytic degradation of ammonia nitrogen: One option is that a series of intermediates such as NH_2_, NH, and N_2_H_x+y_ (*x*, *y* = 0, 1, 2) are generated in the reaction process—they then react into nitrogen molecules. The second option is that NO_2_^−^ and NO_3_^−^ ions are finally generated through HONH_2_ intermediates. The entire reaction is given as follows [16]:

NH_3_ + ·OH → NH_2_ + H_2_O (8)

NH_2_ + ·OH → NH + H_2_O (9)

NH +·OH → N + H_2_O (10)

NH_x_ + NH_y_ → N_2_H_x+y_ (*x*, *y* = 0, 1, 2) (11)

N_2_H_x+y_ + (*x*+*y*)OH → N_2_ + (*x* + *y*)H_2_O (12)

NH_3_ + ·OH → HONH_2_ + H^+^ (13)

HONH_2_ + ·OH → NO_2_^−^ → NO_3_^−^ (14)

## 4. Summary

1. The {001}TiO_2_/TiOF_2_ was prepared with 15 mL hydrofluoric acid and had a large specific surface area to provide more active sites for the reaction. It has a 92.25% highly active {001} crystal surface on which hydroxyl oxygen and oxygen vacancy (Ov) in water is adsorbed. It has a strong optical absorption capacity, small bandwidth, and strong photogenerated carrier separation ability. This indicates that the system has good photocatalytic activity;
2. The volatilization loss rate of ammonia nitrogen increases with increasing pH. When the initial concentration of ammonia nitrogen is 100 mg/L and pH is 10, the degradation rate of ammonia nitrogen by 100 mg F15 is 93.19%. Throughout the entire reaction process, the catalyst itself has little influence on the change in ammonia nitrogen concentration, and photocatalysis plays a major role;
3. Hydroxyl and superoxide radicals are produced during F15 degradation of ammonia nitrogen; their products are mainly N_2_, accompanied by a small amount of NO_2_^−^ and NO_3_^−^.

## Figures and Tables

**Table 1:** Peak intensity and the ratio of the Raman vibrational modes between Eg and A1g.

Samples	Peak Intensity of Eg (514 cm^−1^)	Peak Intensity of A1g (144 cm^−1^)	Percentage of {001}
F6	1096.91	3645.23	30.09%
F9	1129.88	2692.69	52.09%
F12	3864.17	7018.22	55.06%
F15	1565.51	1697.08	92.25%

**Table 2:** Structural parameters of FX.

Samples	Specific Surface Area (m^2^/g)	Pore Volume (cm^3^/g)	Pore Diameter (nm)
F6	52.54	0.14	8.96
F9	56.76	0.16	8.95
F12	57.86	0.15	7.95
F15	60.89	0.14	8.46

## Data Availability

The data that support the findings of this study are available from the corresponding author, upon reasonable request.
